# *Eucommia ulmoides* extract attenuates oxidative stress and promotes melanogenesis via Wnt/β-catenin signaling in B16 cells and mice

**DOI:** 10.55730/1300-0152.2780

**Published:** 2025-09-22

**Authors:** Xiaojin LIU, Yaqian QIU, Xiaobing LV, Lei CHANG, Tiancheng JI, Yefan GU, Shuyue CHEN

**Affiliations:** 1 Anhui Provincial Key Laboratory of Molecular Enzymology and Mechanism of Major Metabolic Diseases, College of Life Sciences, Anhui Normal University, Wuhu, Anhui, China; 2 Auhui Provincial Engineering Research Centre for Molecular Detection and Diagnostics, College of Life Sciences, Anhui Normal University, Wuhu, Anhui, China

**Keywords:** Oxidative stress, *Eucommia ulmoides* extract, melanocyte protection, Wnt/β-catenin signaling, hair follicle pigmentation

## Abstract

**Background/aim:**

Oxidative stress is a major contributor to melanocyte dysfunction and hair graying by impairing key signaling pathways. *Eucommia ulmoides* bark extract (EUE), rich in antioxidant phytochemicals, has shown potential in combating oxidative damage. This study investigated the protective and promelanogenic effects of EUE under hydrogen peroxide (H_2_O_2_)-induced oxidative stress, with a focus on the Wnt/β-catenin signaling pathway.

**Materials and methods:**

An oxidative stress model was established using B16 cells and a C57BL/6 mouse hair follicle model.

**Results:**

EUE significantly improved melanocyte survival and reduced intracellular reactive oxygen species (ROS). Mechanistically, EUE activated the Wnt/β-catenin pathway, leading to upregulation of the microphthalmia-associated transcription factor (MITF) and its downstream melanogenic enzymes (TYR, TRP-1, TRP-2), thereby enhancing tyrosinase activity and restoring melanin synthesis. In vivo, topical application of EUE protected hair follicles from H_2_O_2_-induced depigmentation and promoted follicular pigmentation.

**Conclusion:**

Our results demonstrate that EUE mitigates oxidative stress and promotes melanogenesis primarily by activating the Wnt/β-catenin-MITF signaling axis. These findings provide strong mechanistic evidence supporting EUE as a potential therapeutic strategy for oxidative stress-related hair graying.

## 1. Introduction

Thick, dark hair is widely regarded as a symbol of youth and vitality across cultures, while graying hair often marks aging. Currently, no effective medical treatments exist for repigmenting white hair, leaving chemical hair dyes—some containing carcinogenic compounds—as the primary solution (Nishimura et al., 2002; Nishimura et al., 2005; Bolt and Golka, 2007; Wood et al., 2009; Yale et al., 2020; He et al., 2022). In human hair follicles (HFs), melanogenesis is governed by tyrosinase (TYR), tyrosinase-related protein-1 (TRP-1), and tyrosinase-related protein-2 (TRP-2), all regulated by the microphthalmia-associated transcription factor (MITF) (D’Mello et al., 2016). The Wnt/β-catenin signaling pathway plays a critical role in melanocyte differentiation, survival, and pigmentation by modulating *MITF* and *TYR* expression (Mulholland et al., 2005; Zang et al., 2019).

The concept of oxidative stress, defined as an imbalance between reactive oxygen species (ROS) and antioxidant defenses, was first introduced by Helmut Sies in 1985 (Sies, 1985; Sies et al., 2017). It contributes to melanocyte apoptosis and hair graying, and has been implicated in numerous degenerative conditions. Factors such as psychoemotional stress, inflammation, and environmental challenges elevate oxidative stress, leading to melanocyte apoptosis and hair depigmentation, often termed the “free radical theory of aging” (Melov, 2002; Arck et al., 2006; Jadkauskaite et al., 2017). Hydrogen peroxide (H_2_O_2_) accumulation exacerbates this process, and its reduction has been shown to mitigate cell damage and support hair health (Forman and Zhang, 2021). Importantly, oxidative stress can suppress Wnt/β-catenin signaling, reducing β-catenin stability, impairing MITF activation, and ultimately downregulating melanogenesis, thereby accelerating pigment loss from the follicular niche (Jadkauskaite et al., 2017; Zang et al., 2019).

*Eucommia ulmoides*, documented in the Chinese Pharmacopoeia since the Han Dynasty over 2000 years ago, is a medicinal plant traditionally used in Asia to treat aging-related conditions, including hypertension, diabetes, and osteoporosis (Zhou et al., 2009; He et al., 2014; Huang et al., 2021). *Eucommia ulmoides* bark extract (EUE) is rich in bioactive compounds with antioxidant properties. This study aimed to determine whether EUE can protect melanocytes from H_2_O_2_-induced oxidative stress and restore pigmentation by modulating the Wnt/β-catenin pathway. Using both B16 murine melanoma cells and a mouse hair follicle model, we assessed melanin production, TYR activity, and key melanogenic signaling molecules, providing mechanistic evidence for EUE as a candidate therapeutic for oxidative stress-induced hair depigmentation.

## 2. Materials and methods

### 2.1. Biological materials, chemicals, and reagents

B16 melanoma cells were obtained from Sciencell Research Laboratories (Carlsbad, CA, USA). Cells were cultured in Dulbecco’s Modified Eagle Medium (DMEM; Gibco, Thermo Fisher Scientific, USA) supplemented with 10% fetal bovine serum (FBS; Gibco), 100 U/mL penicillin, and 100 μg/mL streptomycin at 37 °C in a humidified atmosphere containing 5% CO_2_. EUE was sourced from Shanghai Zhina Biotechnology Co., Ltd. CCK-8 was purchased from Yeasen (Shanghai, China). 3-Isobutyl-1-methyl-xanthine (IBMX) was purchased from BOSF Biotechnology Co., Ltd (Hefei, China). L-3,4-dihydroxyphenylalanine (L-DOPA) was purchased from Hefei Bomei Biotechnology Co., Ltd. Enhanced chemiluminescence (ECL) reagent was obtained from Beijing Labgic Technology Co., Ltd. Poly-vinylidene fluoride (PVDF) membranes were supplied by Beyotime Biotech Inc. Antibodies were purchased from Proteintech Group, Inc. Male C57BL/6 mice were purchased from Henan Sikebas Biotechnology Co., Ltd., China. The Leica DMi8 inverted microscope was purchased from Leica Microsystems (Germany), the BD FACSCanto II flow cytometer was purchased from Becton Dickinson (USA), and the Spark 10M multimode microplate reader was purchased from Tecan Group Ltd. (Männedorf, Switzerland; Asset ID: 17104551).

### 2.2. Cell viability assay

Cell viability was assessed using the CCK-8 kit. B16 melanoma cells were seeded in 96-well plates at a density of 1 × 10^4^ cells/well and incubated overnight. Cells were first exposed to 1.5 mM H_2_O_2_ for 0.5 h in the dark at 37 °C, and then treated with EUE (0, 0.1, 0.2, 0.4, or 0.8 mg/mL) for 48 h. Optical density (OD) was measured at 450 nm using a microplate reader. Each experiment was performed in triplicate. The doses and exposure times of EUE and H_2_O_2_ were determined based on our lab’s preliminary cytotoxicity tests, confirming moderate cell viability (~80%) under oxidative stress.

### 2.3. Intracellular ROS measurement

Intracellular ROS levels were quantified using 2,7′-dichlorodihydrofluorescein diacetate (DCFH_2_-DA) staining, as described by Hseu (Hseu et al., 2019). B16 cells were treated with 1.5 mM H_2_O_2_ to induce oxidative stress, followed by the addition of EUE (0.1, 0.2, 0.4, or 0.8 mg/mL). Cells were incubated with DCFH_2_-DA (10 μM) at 37 °C for 30 min in the dark, counterstained with DAPI for 5 min, and analyzed using an inverted fluorescence microscope (200 × magnification) and a flow cytometer.

### 2.4. Measurement of intracellular melanin content

Intracellular melanin content in melanoma cells was measured following established protocols (Huang et al., 2020; Chen et al., 2021; Liu et al., 2024). Cells were exposed to 1.5 mM H_2_O_2_ for 0.5 h to induce oxidative stress. Subsequently, cells were treated for an additional 48 h with either EUE (0.1, 0.2, 0.4, or 0.8 mg/mL) or IBMX (35 μM), a protein kinase inhibitor. A blank control was included without H_2_O_2_, EUE, or IBMX. Posttreatment, cell pellets were collected and solubilized in 1 M NaOH at 60 °C for 60 min. Melanin levels were quantified spectrophotometrically at 405 nm, and absorbance was measured using a multimode microplate reader.

### 2.5. Assay of mushroom TYR and intracellular TYR activity

Mushroom TYR and intracellular TYR activities were assayed following previously described methods (Huang et al., 2020; Chen et al., 2021; Liu et al., 2024). Briefly, melanoma cells were exposed to 1.5 mM H_2_O_2_ for 0.5 h to simulate oxidative stress, followed by treatment with EUE (0.1, 0.2, 0.4, or 0.8 mg/mL) or IBMX (35 μM) for 48 h. A blank control was included without H_2_O_2_, EUE, or IBMX. Posttreatment, cell extracts (100 μL) were mixed with freshly prepared 0.1% L-DOPA solution (in PBS), incubated at 37 °C for 30 min, and the absorbance was measured at 490 nm to assess TYR activity.

### 2.6. Gene expression analysis

Quantitative real-time polymerase chain reaction (qRT–PCR) was performed to evaluate the expression levels of melanogenesis-related genes, including *Wnt5a, β-catenin, MITF, TYR, TRP-1*, and *TRP-2*. All experimental methods, primers, and instruments were adapted from the report by Liu et al. (Liu et al., 2022; Liu et al., 2024).

### 2.7. Western blot analysis

B16 cells were lysed in PBS containing proteinase inhibitors at 4 °C for 20 min. Proteins (30 μg) were separated by sodium dodecyl sulfate–polyacrylamide gel electrophoresis (SDS–PAGE) and transferred to a PVDF membrane. The membrane was blocked with 5% nonfat milk in PBST for 1 h, then incubated overnight at 4 °C with primary antibodies against MITF, TRP1, TRP2, TYR, GSK3β, p-GSK3β, Wnt5a, β-catenin, and β-tubulin. After washing, the membrane was incubated with horseradish peroxidase (HRP)-conjugated goat antimouse secondary antibody (1:7500) at room temperature for 2 h. Protein bands were visualized using ECL reagent and quantified with Multi Gauge 3.0 software, normalized to glyceraldehyde-3-phosphate dehydrogenase (β-tubulin) levels.

Primary antibodies were obtained from Proteintech Group (Chicago, IL, USA): MITF (catalog number 13092-1-AP, 1:1500), TYR (catalog number 31291-1-AP, 1:1000), Wnt5a (catalog number 55184-1-AP, 1:1500), β-catenin (catalog number 51067-2-AP, 1:6000), GSK-3β (catalog number 82061-1-RR, 1:10,000), and p-GSK-3β (catalog number 67558-1-Ig, 1:5000). β-tubulin (catalog number 10094-1-AP, 1:6000) was used as the internal control. The secondary HRP-conjugated goat antimouse IgG antibody (catalog number SA00001-1, 1:7500) was used.

### 2.8. In vivo evaluation of EUE effects on pigmentation under H_2_O_2_-induced stress

The effects of EUE on hair pigmentation under oxidative stress were evaluated using C57BL/6 mice, with all procedures approved by the Academic Ethics Committee of Anhui Normal University (Permit: AHNU-ET2022064). Twenty-seven-week-old healthy male mice (23~25 g) were housed in a temperature-controlled room with a 12-h light/dark cycle and provided food and water ad libitum. The dorsal skin (2 cm × 2 cm) of each mouse was shaved to induce the hair follicle transition from telogen to anagen. Mice were randomly divided into five groups (n = 4 per group):

Negative control: 200 μL of 3% H_2_O_2_ applied at 9 am and 4 pm daily.

Blank control (CK): 200 μL of water applied at 9 am daily.

Three to five treatment groups: 200 μL of 3% H_2_O_2_ applied at 9 am, followed by 200 μL of 0.2% or 0.4% EUE, or 1% 8-MOP (positive control) at 4 pm daily.

Treatments were administered topically to the shaved area once daily for 21 days. Hair pigmentation and growth were subsequently assessed via histological analysis. Following hematoxylin and eosin (H&E) staining, histological sections were imaged under a light microscope. To semiquantitatively assess melanin density, these images were analyzed using ImageJ software (National Institutes of Health, USA)^1^. Briefly, for each hair follicle cross-section, the area containing melanin was manually selected as the region of interest (ROI). The integrated density (IntDen) of the ROI was measured. The average IntDen value of the blank control group was set as 100%, and the data from all other groups were normalized and expressed as a percentage relative to the control.

### 2.9. Statistical analysis

Data were expressed as mean ± standard deviation (SD). Statistical significance was assessed using one-way analysis of variance (ANOVA) followed by Dunnett’s test for pairwise comparisons. Significance levels were defined as *p < 0.05, **p < 0.01, ***p < 0.001 and ***** p < 0.0001.

## 3. Results

### 3.1. Effect of EUE on B16 melanoma cell viability

**Figure 1:**
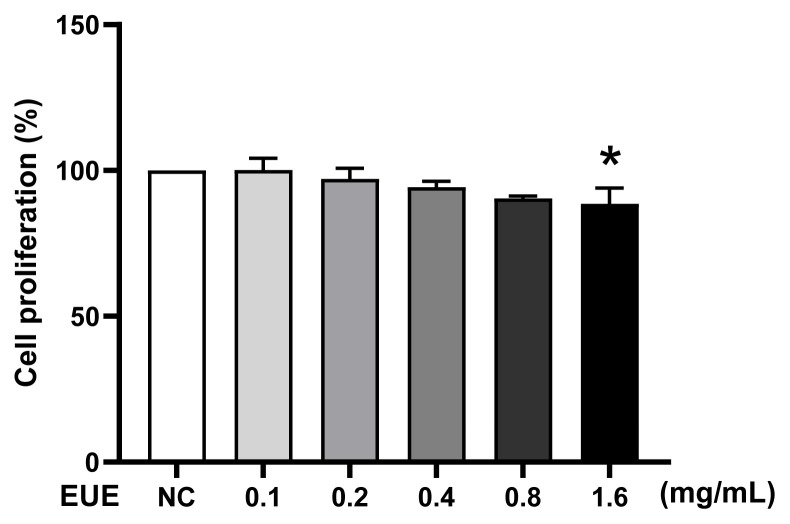
Effect of EUE on B16 cell proliferation. After 24 h of incubation, cell viability was measured using the CCK-8 assay. Data are expressed as a percentage of the viable cell number observed in the NC group, and each column shows the mean values ± SD from three independent experiments performed in triplicate.

The cytotoxicity of EUE on B16 melanoma cells was evaluated using the CCK-8 assay (Figure 1). Cells were treated with EUE (0.1, 0.2, 0.4, 0.8, or 1.6 mg/mL) for 24 h or 48 h. After 24 h, cell viability relative to the negative control (NC) was 97.34%, 94.61%, 92.90%, 89.76%, and 84.78% (*p < 0.05 at 1.6 mg/mL), with similar trends observed at 48 h. Higher concentrations (≥1.6 mg/mL) reduced viability below 85%, indicating mild cytotoxicity. Thus, EUE concentrations below 1.6 mg/mL (i.e. ≤0.8 mg/mL) were selected for subsequent experiments.

### 3.2. EUE attenuates H_2_O_2_-induced oxidative damage in B16 melanoma cells

**Figure 2:**
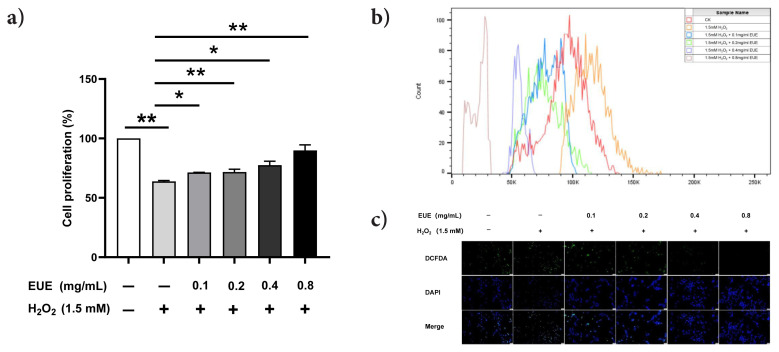
Effect of EUE in reducing oxidative damage. (A) B16 melanocytes with different concentrations of EUE for 48 h before adding 1.5 mM H_2_O_2_; cell viability measured using the CCK-8 assay. (B) Intracellular ROS production was studied using flow cytometry. (C) Intracellular ROS distribution was studied using fluorescence microscopy. Data are reported as mean ± SD. A Student’s t-test was used to compare the data. *p < 0.05, **p < 0.01.

To assess EUE’s protective effect against oxidative stress, B16 melanoma cells were pretreated with EUE (0.1–0.8 mg/mL) for 48 h, followed by 1.5 mM H_2_O_2_ exposure. The CCK-8 assay (Figure 2A) showed that EUE mitigated H_2_O_2_-induced cytotoxicity, with 0.8 mg/mL yielding the greatest protection. Flow cytometry (Figure 2B) revealed elevated intracellular ROS levels after H_2_O_2_ treatment compared with the control (CK), and EUE reduced these levels in a dose-dependent manner. Fluorescence microscopy with DCFH_2_-DA staining (Figure 2C) revealed strong green fluorescence intensity after H_2_O_2_ exposure, indicating elevated ROS levels. EUE treatment reduced fluorescence intensity in a dose-dependent manner, suggesting effective ROS scavenging.

### 3.3. Effect of EUE on melanin content in B16 melanoma cells

**Figure 3:**
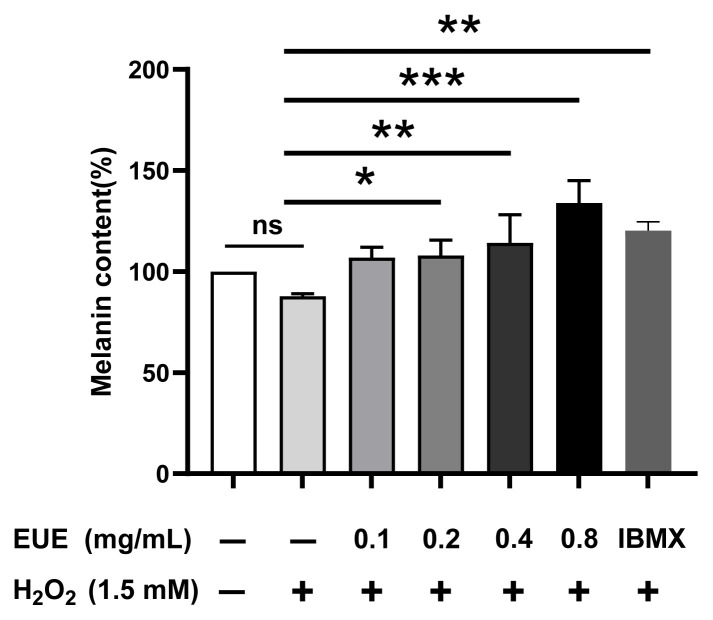
Melanin content was quantified using the NaOH method. *p < 0.05; **p < 0.01, ***p < 0.001. ns, not significant.

EUE treatment dose-dependently increased melanin content in B16 melanoma cells under 1.5 mM H_2_O_2_-induced oxidative stress (Figure 3). After 24 h, melanin levels at EUE concentrations of 0.1, 0.2, 0.4, and 0.8 mg/mL were 101.02%, 110.46% (*p < 0.05), 124.81% (**p < 0.01), and 132.88% (***p < 0.001) relative to the H_2_O_2_-treated group, respectively. Compared with the positive control IBMX (35 μM), 0.8 mg/mL EUE yielded 120.20% (**p < 0.01) of the melanin content, while H_2_O_2_ alone reduced it to 89.03% of the untreated baseline. These results indicate that EUE (>0.1 mg/mL) counteracted H_2_O_2_-induced depigmentation, with higher doses (0.4 and 0.8 mg/mL) surpassing IBMX in stimulating melanogenesis.

### 3.4. Effects of EUE on mushroom and intracellular TYR activity in B16 melanoma cells

**Figure 4:**
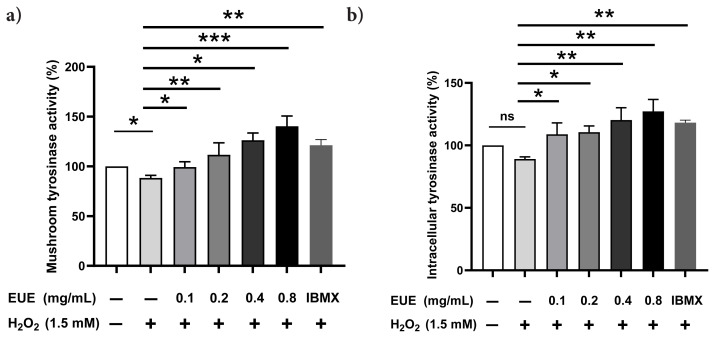
Effect of EUE on the activity of mushroom TYR (A) and intracellular TYR (B).

EUE treatment significantly enhanced both mushroom and intracellular TYR activities in B16 melanoma cells under 1.5 mM H_2_O_2_-induced oxidative stress (Figure 4). For mushroom TYR (Figure 4A), 24 h treatment with EUE at 0.1, 0.2, 0.4, and 0.8 mg/mL increased activity to 105.40% (*p < 0.05), 125.22% (**p < 0.01), 134.58% (*p < 0.05), and 152.21% (***p < 0.001) relative to the H_2_O_2_-treated group, respectively, compared with 121.41% (**p < 0.01) for IBMX (35 μM). H_2_O_2_ alone reduced activity to 91.29% (*p < 0.05) of the negative control (NC). For intracellular TYR (Figure 4B), EUE at the same concentrations elevated activity to 117.59% (*p < 0.05), 115.81% (*p < 0.05), 124.97% (***p < 0.001), and 129.93% (****p < 0.0001) relative to the H_2_O_2_ group, compared with 115.81% (**p < 0.01) for IBMX, with H_2_O_2_ reducing activity to 90.21% (*p < 0.05) of NC. IBMX served as a positive pigmentation standard, while H_2_O_2_ acted as a depigmentation control. These results demonstrate that EUE (>0.1 mg/mL) counteracted H_2_O_2_-induced inhibition, with the highest dose (0.8 mg/mL) outperforming IBMX in stimulating both TYR activities.

### 3.5. EUE upregulates melanin synthesis-related gene expression in B16 cells

**Figure 5:**
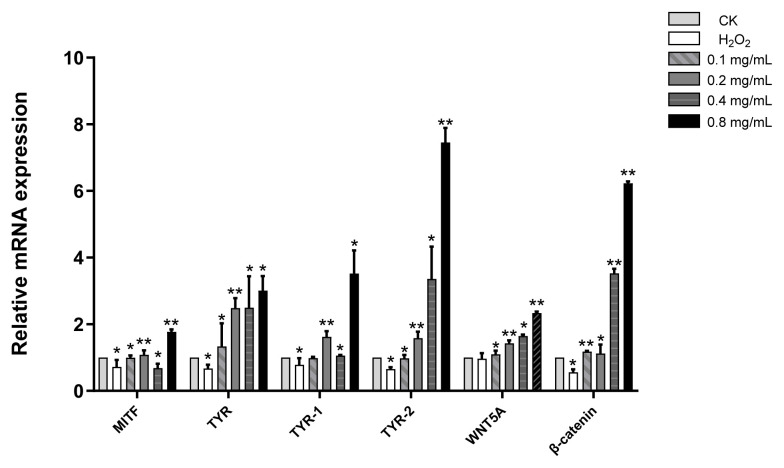
Effect of EUE on the expression of six melanogenic genes after H_2_O_2_ exposure.

In the anagen phase of HFs, melanocytes produce melanin and transfer it to keratinocytes via melanosomes, a process regulated by MITF and the Wnt/β-catenin pathway (Paus et al., 2013; Huang et al., 2020). To assess EUE’s effect on this pathway, mRNA levels of six melanin synthesis genes in B16 melanoma cells were measured by qRT–PCR under 1.5 mM H_2_O_2_ stress (Figure 5). After 25 min of H_2_O_2_ exposure, messenger ribonucleic acid (mRNA) levels decreased to *MITF* (0.72), *TYR* (0.67), *TRP-1* (0.78), *TRP-2* (0.65), *Wnt5a* (0.97), and *β-catenin* (0.56) relative to the blank control. Treatment with EUE (0.1, 0.2, 0.4, or 0.8 mg/mL) for 24 h dose-dependently increased the mRNA levels compared with the negative control (NC), with fold changes peaking at 0.8 mg/mL: *MITF* (1.77), *TYR* (3.00), *TRP-1* (3.52), *TRP-2* (7.45), *Wnt5a* (2.33), and *β-catenin* (6.23). These findings suggest that EUE promotes melanogenesis by activating the Wnt/β-catenin signaling pathway under oxidative stress.

### 3.6. EUE enhances melanogenesis-related and signalling protein expression in B16 cells

**Figure 6:**
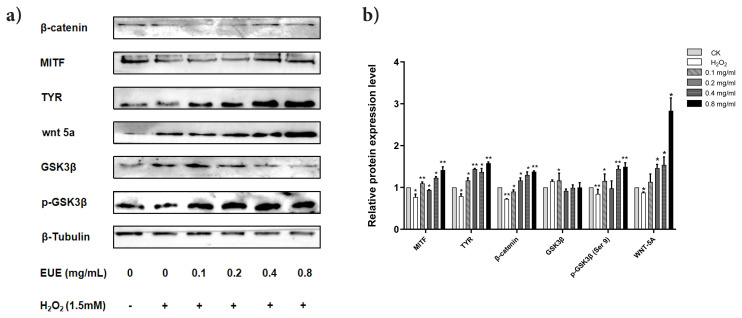
Effects of EUE on melanogenesis-related protein expression in B16 cells. (A) Western blot of MITF, TYR, Wnt-5a, β-catenin, GSK3β, and p-GSK-3β. β-tubulin was used as a control. (B) Bar graphs showing quantitative analysis of protein expression by ImageJ. Data are expressed as mean ± SD (n = 3). *p < 0.05, **p < 0.01 vs. control group.

Western blot analysis showed that EUE treatment under 1.5 mM H_2_O_2_ stress increased the expression of MITF, TYR, β-catenin, GSK-3β, p-GSK-3β, and Wnt-5a in B16 melanoma cells (Figure 6). After 24 h treatment with EUE (0, 0.1, 0.2, 0.4, or 0.8 mg/mL), protein levels relative to the control were MITF (0.77, 1.10, 0.94, 1.23, 1.42), TYR (0.79, 1.17, 1.44, 1.37, 1.58), β-catenin (0.73, 0.90, 1.17, 1.30, 1.38), GSK-3β (1.05, 1.12, 0.92, 0.99, 1.00), p-GSK-3β (0.85, 1.16, 0.98, 1.45, 1.49), and Wnt-5a (0.88, 1.14, 1.46, 1.54, 2.83). The stimulatory effect was most pronounced at 0.8 mg/mL, with significant upregulation of MITF, TYR, β-catenin, GSK-3β, p-GSK-3β, and Wnt-5a, while GSK-3β levels remained unchanged. These findings suggest that EUE promotes melanogenesis via the Wnt/β-catenin pathway under oxidative stress, with minimal impact on GSK-3β signalling. Protein expression patterns in Figure 6A and Supplementary Figure were consistent with the corresponding densitometric analysis in Figure 6B, and the trends were reproducible across three independent experiments.

### 3.7. Hair growth and histological analysis in EUE-treated depilated mice

**Figure 7:**
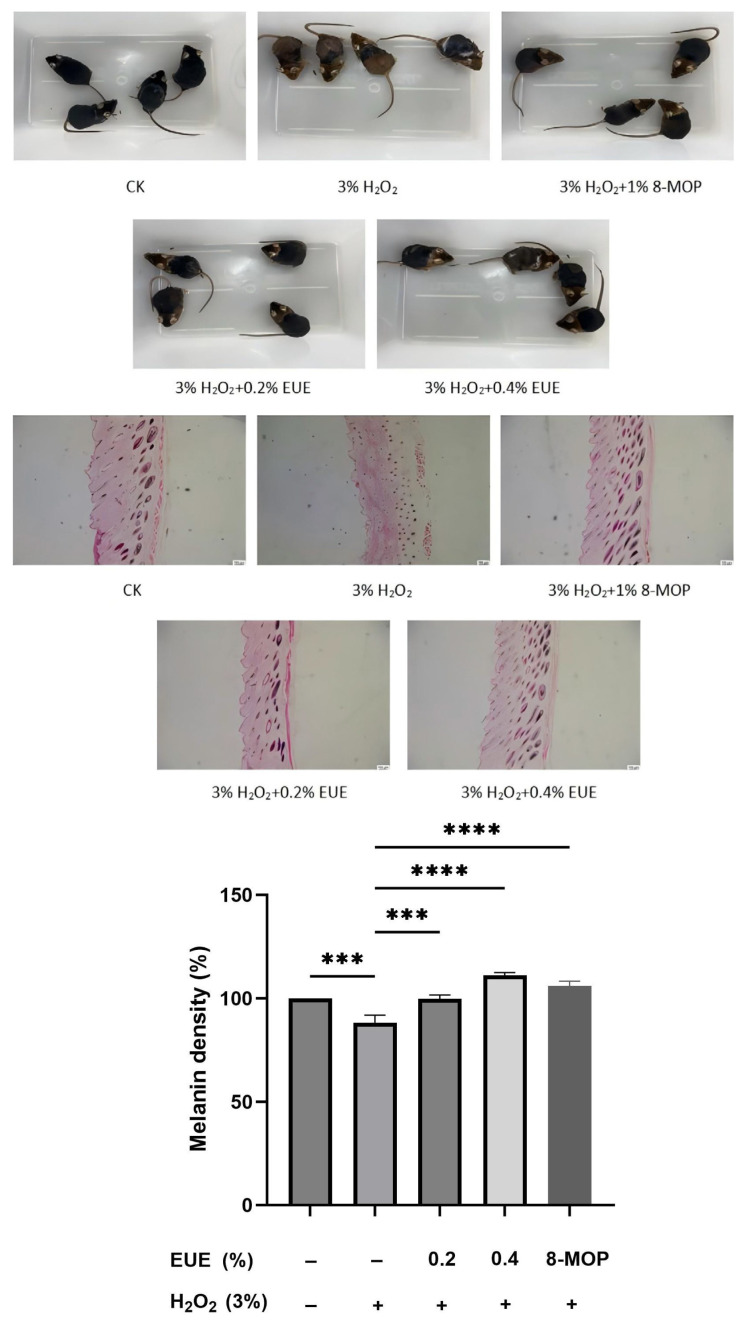
EUE attenuates H_2_O_2_-induced hair depigmentation in C57BL/6 mice. (A) Photographs of the treated C57BL/6 mice after 21 days. (B) Histological examination of hair follicle cross-sections (H&E staining). The scale bar represents 100 μm. (C) Semiquantitative analysis of melanin density in hair follicles. Melanin density was measured from histological images using ImageJ and expressed relative to the blank control group (set at 100%). Data are presented as mean ± SD (n = 4). Statistical significance was determined by one-way ANOVA with Dunnett’s post hoc test.

Hair growth and pigmentation in EUE-treated C57BL/6 mice were evaluated via photography under 3% H_2_O_2_-induced oxidative stress (Figure 7A). The H_2_O_2_ group exhibited yellow hairs in pigment islands, indicating depigmentation, whereas the EUE/H_2_O_2_ and 8-MOP (positive control) groups showed black hairs, suggesting EUE counteracted H_2_O_2_-induced depigmentation. H&E staining of dorsal skin sections further revealed that H_2_O_2_ reduced melanin content in HFs and shafts, while EUE treatment increased melanin staining compared with the H_2_O_2_-treated controls (Figure 7B). To provide quantitative support for these histological observations, we performed a semiquantitative analysis of melanin density in hair follicle cross-sections using ImageJ. The results demonstrated that H_2_O_2_ treatment significantly reduced follicular melanin density compared with the blank control group (**p < 0.0001). Importantly, topical application of both 0.2% and 0.4% EUE effectively attenuated this H_2_O_2_-induced reduction in a dose-dependent manner (***p < 0.001 and ****p < 0.0001, respectively), with the higher concentration of EUE (0.4%) even elevating melanin levels above baseline. The positive control, 8-MOP, also exhibited a significant restorative effect. These quantitative data, presented in Figure 7C, strongly corroborate our visual findings and confirm the potent protective effect of EUE against oxidative stress-induced hair follicle depigmentation. By day 21, EUE-treated mice displayed faster hair growth and thicker skin than the H_2_O_2_ group, trends consistent with enhanced follicular health. These findings indicate EUE’s potential to promote hair growth and pigmentation, warranting further investigation into its efficacy for the treatment of androgenetic alopecia.

## 4. Discussion

Oxidative stress is a fundamental driver of hair graying through ROS-mediated damage to melanocytes and disruption of regulatory networks that sustain pigmentation (Nishimura et al., 2002; Van Neste and Tobin, 2004; Nishimura et al., 2005; Paus et al., 2013; Liu et al., 2022; Zhang et al., 2022). In this study, EUE protected B16 melanocytes from H_2_O_2_-induced injury and preserved follicular pigmentation in a mouse model. Beyond its antioxidant capacity, EUE increased melanin content and TYR activity, and upregulated key melanogenic regulators, including MITF, TRP-1, and TRP-2.

Notably, EUE elevated β-catenin levels and increased GSK-3β phosphorylation, a modification known to reduce β-catenin degradation. This suggests that EUE may inhibit β-catenin turnover, thereby sustaining melanocyte activity. This is in agreement with reports showing that GSK-3β inhibition promotes melanogenesis (Zang et al., 2019), and it supports a mechanistic model in which EUE stabilizes β-catenin and promotes MITF-dependent transcription of melanogenesis genes under oxidative stress.

These findings extend prior reports that focused primarily on the antioxidative properties of *E. ulmoides* by providing functional and mechanistic evidence for modulation of the Wnt/β-catenin-GSK-3β axis under oxidative challenge (Cheng et al., 2022; Park et al., 2025). Regarding phytochemical composition, EUE contains multiple bioactive constituents, including geniposidic acid, lignans, and flavonoids, which have been reported to exert antioxidant and melanogenesis-modulating effects. These components may act synergistically to confer the observed protective outcomes. The dual action—ROS attenuation and signaling restoration—suggests EUE may both reduce oxidative burden and reestablish propigmentary signaling, which is particularly relevant for maintaining melanocyte stem cell function within the follicular niche.

Limitations of the study include the modest in vivo sample size (n = 4 per group), which may reduce statistical power. The present findings should therefore be interpreted as preliminary evidence. Nonetheless, consistent trends were observed across groups, supporting the reliability of the outcomes. Future studies with larger cohorts are warranted to validate these results and enhance the robustness of the conclusions. Additionally, the complex, multicomponent nature of EUE presents both a challenge and an opportunity for future research. While our findings are promising, further validation in human scalp melanocytes and hair follicle organ cultures is necessary to assess translational potential. Additionally, comprehensive safety profiling, skin penetration studies, and formulation optimization will be essential before considering clinical applications. Future studies should (i) isolate and characterize bioactive constituents, (ii) validate effects in primary human scalp melanocytes and human hair follicle organ cultures, and (iii) employ pathway-specific perturbations to establish causality. Additionally, formulation development, penetration studies, and long-term safety profiling will be required before clinical translation. Overall, our data provide a mechanistic basis for further development of EUE-derived strategies to mitigate oxidative stress-related hair depigmentation.

## Supplementary material

Supplementary Figure Original uncropped western blot images corresponding to Figure 6A. This figure shows the uncropped original western blot bands for MITF, YR, β-catenin, GSK3β, p-GSK3β, Wnt5a, and β-tubulin.

## Data Availability

The data reported in this study’s findings are not publicly available due to commercial agreements. However, upon a reasonable request, the data can be made available from the corresponding author. Further inquiries can be directed to the corresponding author.

## Footnotes

1 ImageJ (2023). ImageJ [online]. Website https://imagej.net/ij/ [accessed 17 September 2025].
