# Supplementary figures and images for: Eucommia ulmoides extract attenuates oxidative stress and promotes melanogenesis via Wnt/β-catenin signaling in B16 cells and mice
Source: Turk J Biol. 2025 Sep 22;49(7):790–9. doi: 10.55730/1300-0152.2780 (PMC12768441; doi:10.55730/1300-0152.2780)

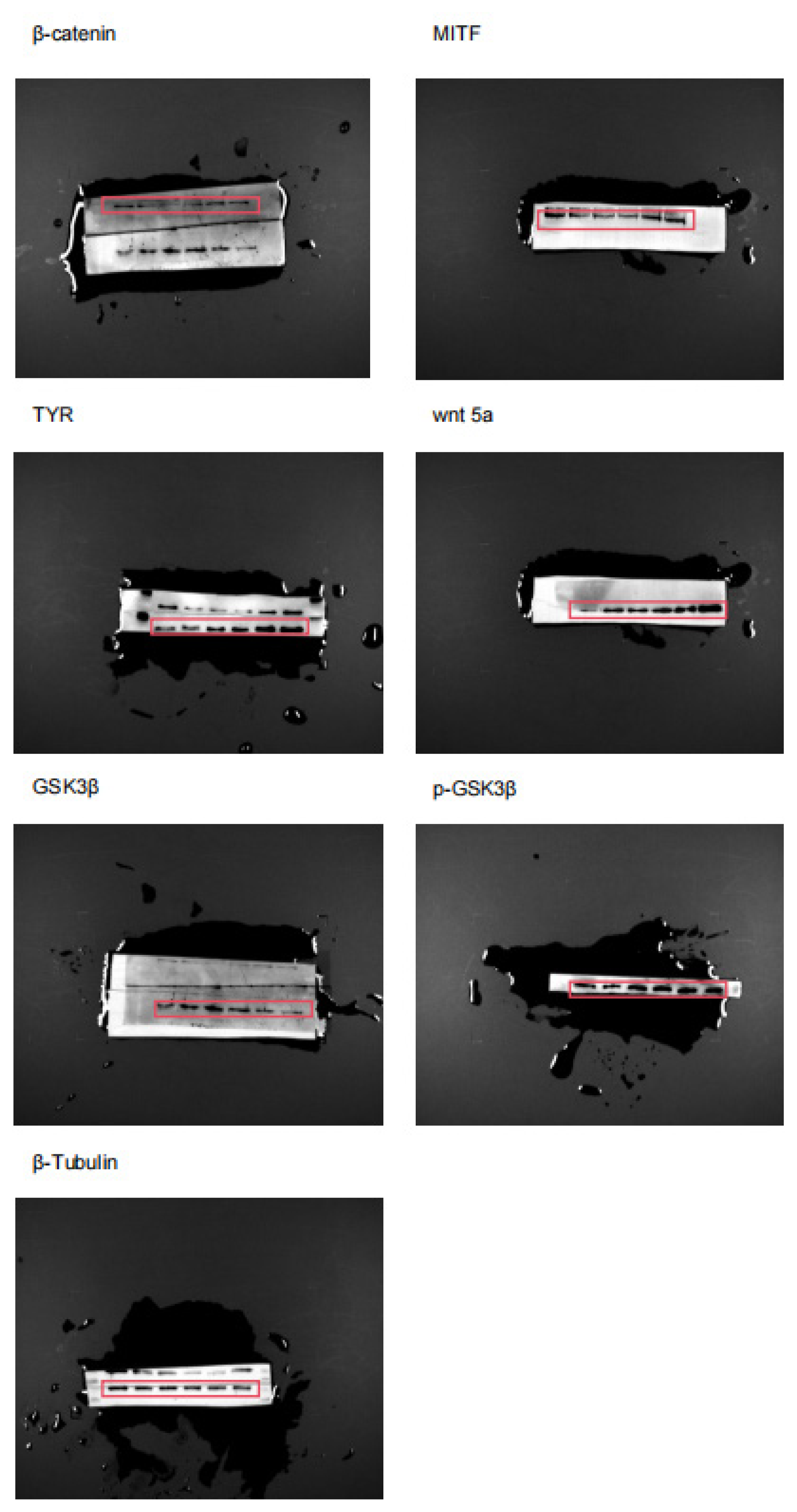

Supplement: Supplementary Figure — Original uncropped western blot images corresponding to Figure 6A. This figure shows the uncropped original western blot bands for MITF, YR, β-catenin, GSK3β, p-GSK3β, Wnt5a, and β-tubulin. [file tjb-49-07-790s1.tif]
